# The Current Situation of Neurological Health in Bangladesh: A Perspective

**DOI:** 10.1002/hsr2.70530

**Published:** 2025-03-02

**Authors:** Mst. Mohona Khatun, Mohammad Shahangir Biswas, Suronjit Kumar Roy, Md Foyzur Rahman, Rubait Hasan, Syed Masudur Rahman Dewan, Munna Kumar Podder

**Affiliations:** ^1^ Department of Biochemistry and Biotechnology Khwaja Yunus Ali University Sirajganj Bangladesh; ^2^ Department of Public Health Daffodil International University Dhaka Bangladesh; ^3^ Department of Biochemistry and Biotechnology University of Science & Technology Chittagong (USTC) Chittagong Bangladesh; ^4^ Department of Pharmacy School of Life Sciences United International University Dhaka Bangladesh

**Keywords:** Bangladesh, depression, mental health, neurological disease, stroke

## Abstract

**Background and Aims:**

In Bangladesh, neurological disorders are becoming a bigger public health concern because they significantly increase disability, mortality, and medical costs. This review explores the current neurological health landscape in Bangladesh, with a focus on prevalent disorders such as stroke, epilepsy, Parkinson's disease, and meningitis.

**Methods:**

To complete this review, we retrieved pertinent information from published articles that we located in Google Scholar, PubMed, and Scopus. We looked up terms like meningitis, stroke, Parkinson's disease, epilepsy, and neurological disease.

**Results:**

Due to common risk factors like diabetes, high blood pressure, and lifestyle choices, the prevalence of these conditions is increasing. Inadequate healthcare infrastructure, especially in rural areas, and a lack of specialized medical care make diagnosis and treatment extremely difficult. Effective disease management is made more difficult by systemic flaws in the public health system and socioeconomic disparities.

**Conclusion:**

Public education campaigns, preventive measures, better access to necessary medications, and improvements to the healthcare infrastructure are all vital to lessen this burden. To improve neurological health outcomes in Bangladesh, this review emphasizes the urgent need for focused interventions and strong policies. It also emphasizes the significance of ongoing research and medical advancements in managing and lowering the prevalence of major neurological disorders in Bangladesh.

## 1 Introduction

Neurological diseases have a major impact on disability and health worldwide. Neurological diseases have a significant impact on disability and global health, as evidenced by their third‐highest death rate in the European Union [1]. These conditions increase the risk of suicide and reduce people's quality of life. A decline in cognitive function is linked to brain microbleeds caused by hypertension [2, 3]. A significantly lower life expectancy is linked to mental, neurological, and drug use disorders (MNSDs), and excess mortality is a significant cause for concern [4]. The high lifetime risk of dementia, stroke, and parkinsonism underscores the necessity of preventative measures, as one in three men and one in two women may experience these conditions in their lifetimes [5]. The burden of disability‐adjusted life years in Spain is mostly increased by neuropsychiatric illnesses, including anxiety, depression, Alzheimer's, migraines, and substance addiction disorders [6]. In the United States, the prevalence of neurological disorders varies at the state, regional, and even national levels [7, 8]. Healthcare providers must regularly check patients and put preventive measures in place because neurological conditions like epilepsy, Parkinson's disease, stroke, and dementia make it much more likely for seniors living in the community to fall [9].

Global health and disability are greatly impacted by neurological diseases. They are a major public health concern in Bangladesh, impacting individuals of all ages and significantly lowering quality of life. Modern allopathic medicine is replacing traditional Ayurvedic therapy, but the healthcare system is still lacking. A major development in the nation's neurological healthcare system occurred in 2012 with the opening of the National Institute of Neuroscience and Hospital in Dhaka. There are only 60 practicing neurologists in the country, and only two medical schools offer post‐graduate training, indicating that there is still a neurology shortage [10]. In Bangladesh, prevalent risk factors like tobacco use, poor food, physical inactivity, and chronic conditions like diabetes and hypertension are contributing to an increase in the incidence of non‐communicable diseases (NCDs), including neurological disorders. NCDs are starting to cluster, which is concerning since it indicates that neurological illnesses will soon become more prevalent. In fact, 38% of people have more than one risk factor [11]. A specific issue in the nation that primarily impacts young male laborers is cervical spinal cord injuries (CSCI), which are caused by falls that occur during the process of carrying heavy objects onto the head. The majority of these injuries cause complete neurological impairments, most commonly affecting the C5 and C6 spinal levels [12]. *Campylobacter jejuni* infection is associated with Guillain−Barré syndrome (GBS), a neurological illness that affects 57% of people in Bangladesh and is associated with high death and severe disability. Positive serology tests for GBS are available [13]. Rural communities are often less informed about diabetes, glaucoma, and age‐related macular degeneration. They also have different habits and levels of awareness when it comes to common eye conditions. There are several reasons for this discrepancy, such as age, low socioeconomic status, and insufficient education [14]. Efforts to promote mental health were launched in Bangladesh amid the COVID‐19 epidemic, with a focus on neurological health and how it plays a role in reducing global mental health issues [15]. Genetic determinants are instrumental in the pathogenesis of neurological disorders, profoundly influencing the comprehension, diagnosis, and therapeutic management of these ailments. In Bangladesh, a nation characterized by a substantial incidence of consanguinity and distinctive genetic heterogeneity, the investigation of neurodevelopmental disorders (NDDs), including autism spectrum disorder (ASD), intellectual disability (ID), and epilepsy, has yielded significant genetic revelations. Sophisticated genomic methodologies such as chromosomal microarray (CMA), exome sequencing (ES), and long‐read genome sequencing have elucidated pathogenic and novel variants that are specific to the Bangladeshi demographic [43]. Notwithstanding advances in this field, genetic research within this locale continues to be inadequately represented, highlighting the imperative for comprehensive genetic testing to enhance early diagnostic capabilities and personalized therapeutic strategies, thereby mitigating the escalating burden of neurological disorders within the nation [44]. This review explores the burden of neurological illness in Bangladesh, focusing on conditions like stroke, epilepsy, Parkinson's disease, and meningitis, and calls for immediate action to address healthcare disparities and improve public health outcomes.

## 2 Epidemiology and Prevalent Neurological Diseases in Bangladesh

The most prevalent neurological conditions in Bangladesh include meningitis, Parkinson's disease, epilepsy, and stroke. Each of these illnesses contributes significantly to the nation's overall neurological disease burden and presents serious public health challenges.

Stroke is one of the leading causes of disability and mortality in Bangladesh. It arises from a lack of oxygen and vital nutrients for brain tissue caused by either reduced or obstructed blood flow to a specific area of the brain. Approximately 145.3 new occurrences of stroke occur within a particular population over a certain time frame, according to Bangladesh's incidence rate of stroke, which is measured per 100,000 people annually. At any one time, the overall number of stroke cases in the population is represented by the prevalence rate of stroke in Bangladesh, which is approximately 584.9 cases per 100,000 people [22]. Recent research conducted in Bangladesh demonstrates the staggering burden of neurological disorders in that country. A study conducted by Chittagong Medical College & Hospital indicates that stroke is the most prevalent neurological condition, accounting for 74% of patients that require hospitalization [16].

A neurological condition known as epilepsy is characterized by recurring seizures that have no apparent cause. An individual's mental health and social interactions may be significantly impacted by these seizures.

The annual rate of new epilepsy diagnoses in Bangladesh is roughly 58.7 cases per 100,000 people annually. The overall number of people living with epilepsy at any given moment is reflected in the prevalence rate, which is around 412.3 instances per 100,000 people [23].

Parkinson's disease is a neurological condition that causes movement impairments. It frequently begins with mild symptoms, including a little tremor in one hand. Over time, these symptoms progressively get worse. The number of new cases diagnosed with Parkinson's disease each year in Bangladesh is roughly 9.8 cases per 100,000 persons annually. The overall number of people with Parkinson's disease at any given time is represented by the prevalence rate, which is around 85.6 cases per 100,000 people [41].

The disease known as meningitis causes inflammation of the membranes that surround the brain and spinal cord. Numerous pathogens, including bacteria, viruses, and fungi, might be the source of this inflammation. Meningitis has an incidence rate of about 12.5 cases per 100,000 persons annually, which is the number of new cases that are identified within a particular population in a given amount of time. The overall number of cases (both new and pre‐existing) within a given population at a given point in time is indicated by the prevalence rate of meningitis, which is approximately 34.7 cases per 100,000 people [24].

Table 1 provides a summary, based on current research and medical reports, of the incidence and prevalence rates of major neurological illnesses in Bangladesh.

**Table 1:** The incidence and prevalence rates of major neurological disorders in Bangladesh.

Disorder	Incidence rate (cases per 100,000/year)	Prevalence rate (cases per 100,000 population)
Stroke	145.3	584.9
Epilepsy	58.7	412.3
Parkinson's disease	9.8	85.6
Meningitis	12.5	34.7

**Figure 1:**
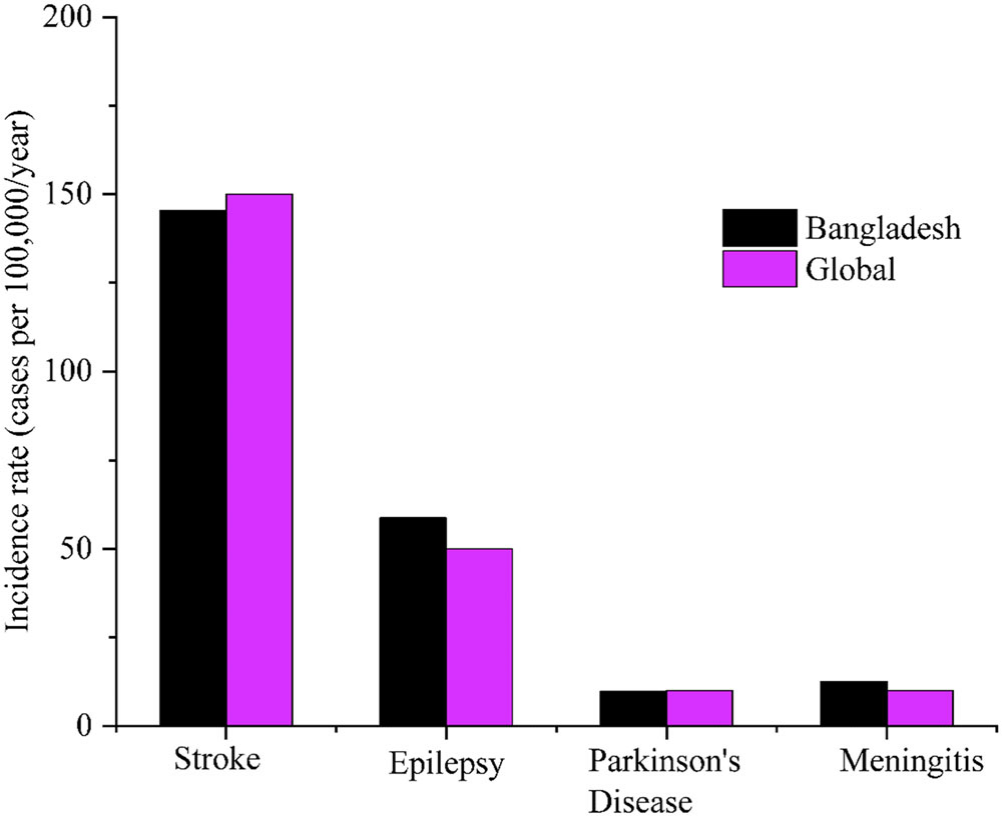
Comparison of incidence rates of neurological disorders: Global versus Bangladesh.

**Figure 2:**
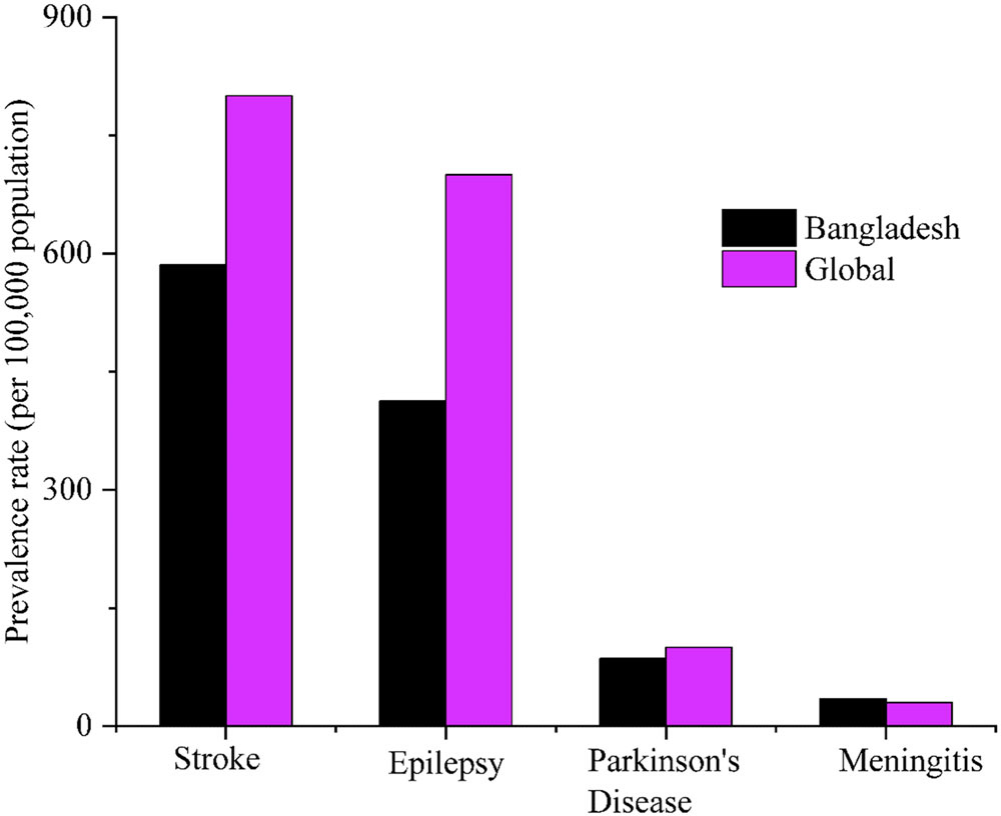
Comparison of prevalence rates of neurological disorders: Global versus Bangladesh.

Figures 1 and 2 compares the incidence and prevalence rates of four neurological disorders—stroke, epilepsy, Parkinson's disease, and meningitis—between the global and Bangladeshi populations.

It highlights that stroke has the highest rates in both regions, followed by epilepsy. Parkinson's disease and meningitis have lower rates overall. Notably, Bangladesh shows higher prevalence rates for all disorders except stroke, where the global incidence rate is higher [22]. According to the same survey, Table 2 shows that, with an average age of 55 years, 74% of neurological patients come from rural areas, whereas about 26% come from urban areas. 53% of patients are male, and 47% are female, indicating a well‐balanced gender distribution [17].

**Table 2:** Demographic and gender distribution of neurological patients by region.

Category	Subcategory	Percentage (%)
Residence	Rural	74
	Urban	26
Gender	Male	53
	Female	47

These statistics demonstrate the substantial influence these illnesses have on public health in Bangladesh by showing both the frequency of new cases and the total number of instances of each ailment that are currently present within the population.

## 3 Risk Factors and Causes

Neurological illnesses in Bangladesh have many different causes and risk factors, including lifestyle, environmental, and hereditary factors. In Bangladesh, neurological problems are influenced by a combination of genetic predispositions and environmental variables such as air pollution, heavy metals, and maybe ambient radiation [18, 19]. Lifestyle factors like exercise, cigarette use, and nutrition are also crucial. These results emphasize the necessity for targeted interventions and preventive strategies to address the specific challenges, given Bangladesh's environmental and lifestyle context [20].

Table 3 summarizes the risk factors for neurological illness in Bangladesh from 2019 to 2023. The enumerated risk determinants encompass elevated systolic blood pressure, increased body mass index (BMI), augmented fasting plasma glucose concentrations, the prevalence of tobacco use, and the presence of ambient particulate matter pollution, all of which play a pivotal role in the onset and advancement of neurological disorders [48]. Throughout the quinquennial span, there has been a gradual escalation in the incidence of the majority of risk factors. Elevated systolic blood pressure evidenced a consistent increase from 55.54% in 2019 to 57.50% in 2023. In a comparable manner, the high BMI surged from 20.86% in 2019 to 22.00% in 2023, while elevated fasting plasma glucose levels ascended from 20.66% to 22.50% within the same period. Conversely, the prevalence of smoking exhibited a modest decrement, declining from 15.65% in 2019 to 15.20% in 2023. The pollution from ambient particulate matter manifested a persistent upward trajectory, rising from 3.80% in 2019 to 4.00% in 2023 [22]. These statistics illuminate the escalating burden of modifiable risk factors that contribute to neurological afflictions in Bangladesh. The increasing prevalence of hypertension, obesity, and disrupted glucose metabolism signifies mounting public health challenges, whereas the reduction in smoking prevalence indicates some advancement in tobacco regulation. Nonetheless, the continual uptick in air pollution accentuates the necessity for environmental interventions aimed at alleviating its adverse effects on neurological well‐being.

**Table 3:** Trends in risk factors for major neurological diseases in Bangladesh (2019−2023).

Year	High systolic blood pressure (%)	High BMI (%)	High fasting plasma glucose (%)	Smoking (%)	Ambient particulate matter pollution (%)
2019	55.54	20.86	20.66	15.65	3.80
2020	56.00	21.10	21.00	15.50	3.85
2021	56.50	21.30	21.50	15.40	3.90
2022	57.00	21.60	22.00	15.30	3.95
2023	57.50	22.00	22.50	15.20	4.00

## 4 Genetic Basis of Neurological Illness in Bangladesh

The genetic foundations of neurological disorders in Bangladesh are undergoing rigorous investigation, with particular emphasis on conditions including NDDs, ASD, lysosomal storage disorders (LSD), and Wilson disease. These explorations elucidate the influence of genetic polymorphisms and their clinical implications within the Bangladeshi demographic. Investigations concerning NDD have delineated copy number variations (CNVs) as pivotal factors contributing to these conditions. Among Bangladeshi pediatric populations, pathogenic CNVs were identified in 12.26% of instances, exhibiting a markedly elevated prevalence among females. Notable genes, including PSMC3 and KMT2B, have been associated with potential correlations to autism and ID, respectively. This emphasizes the critical role of genetic diagnostics in comprehending these disorders [44]. In relation to ASD, research has demonstrated a robust correlation between the CNTNAP2 gene variant (rs7794745) and ASD among Bangladeshi children. This genetic variation correlates with pronounced challenges in social interactions, language deficits, and behavioral irregularities, indicating its potential as a biomarker for early diagnosis and intervention [45]. LSD, transmitted through autosomal recessive or X‐linked inheritance patterns, were also scrutinized within a Bangladeshi cohort. Mucopolysaccharidosis and metachromatic leukodystrophy emerged as the most frequently encountered forms. Clinical presentations encompassed neuroregression, microcephaly, and dysmorphic features, often accompanied by significant alterations observable via neuroimaging techniques [46]. Wilson disease, a hereditary condition characterized by disrupted copper metabolism, has been investigated concerning its neuropsychiatric manifestations in Bangladeshi children. Symptoms such as alterations in behavior, depressive states, and anxiety were prevalent, with neuroimaging consistently revealing hyperintensity within the basal ganglia. These observations underscore the genetic foundations and clinical intricacies of neurological disorders in Bangladesh, underscoring the necessity for improved diagnostic and therapeutic strategies [47].

## 5 Clinical Features and Diagnostic Criteria

In Bangladesh, neurological illnesses place a heavy strain on the healthcare system and need prompt diagnosis and suitable therapy. However, effective management is sometimes hampered by limited access to healthcare services, particularly in rural regions. The clinical characteristics, course, and diagnostic standards for meningitis, stroke, epilepsy, and Parkinson's disease are described in the discussion that follows.

There are two forms of stroke: ischemic and hemorrhagic. Sudden numbness or weakness, trouble speaking, disorientation, visual abnormalities, and loss of balance are all signs of an ischemic stroke [25]. Hemorrhagic stroke, on the other hand, usually manifests as a strong headache, nausea, vomiting, and unconsciousness [26]. The severity and recovery outcomes of strokes depend on the damaged brain area and prompt management. Stroke progression can cause long‐term impairments such as paralysis, speech problems, and memory loss [27]. Clinical examination using the FAST criteria, imaging modalities such as CT/MRI to differentiate between different forms of stroke, blood tests to identify risk factors, and ECG or echocardiography to assess heart‐related causes are among the diagnostic criteria used in Bangladesh [28].

Seizures, which can be localized or generalized tonic‐clonic (convulsions), are a hallmark of epilepsy [29]. Before a seizure, patients may also have an aura, which includes abnormal feelings or alterations in their senses [31]. Untreated epilepsy can worsen a patient's quality of life by causing more frequent and severe episodes [30]. A thorough clinical history of seizure type, frequency, and causes is necessary for diagnosis. Neuroimaging, such as MRI or CT scans, is also necessary to reveal structural abnormalities, and EEG is used to detect aberrant brain activity [32].

In addition to non‐motor symptoms including mood problems, sleep difficulties, autonomic dysfunction, and cognitive loss, Parkinson's disease manifests as motor symptoms such tremor, bradykinesia, stiffness, and postural instability [33]. Symptoms gradually increase as the disease advances, frequently resulting in severe impairment. Clinical evaluation, which assesses motor symptoms and the response to dopaminergic medication, is the basis for diagnosis. Imaging methods such as DaTscan are sometimes employed to verify dopamine depletion [34].

The signs and symptoms of meningitis vary depending on the kind. The symptoms of viral meningitis are often milder and flu‐like, but those of bacterial meningitis include rapid fever, headache, neck stiffness, photophobia, nausea, and vomiting [35]. Rapid progression of bacterial meningitis can result in serious complications or death, whereas viral meningitis often has a longer recovery period and a better prognosis [36]. Lumbar puncture for cerebrospinal fluid analysis, blood cultures to detect bacterial infections, and imaging methods such as CT or MRI to evaluate complications or validate suspected cases are among the diagnostic criteria [35]. A summary of the clinical characteristics, symptoms, progression, and diagnostic standards of prevalent neurological illnesses in Bangladesh is provided in the following Table 4.

**Table 4:** Clinical features, progression, and diagnostic criteria for major neurological diseases in Bangladesh.

Neurological disease	Clinical features and symptoms	Progression	Diagnostic criteria used in Bangladesh
Stroke	Ischemic stroke: Sudden numbness/weakness, confusion, difficulty speaking, visual disturbances, loss of balance [25]. Hemorrhagic stroke: Severe headache, nausea, vomiting, loss of consciousness [26].	Leads to long‐term disabilities (paralysis, speech problems, memory loss). Severity and recovery depend on the brain area affected and timely intervention [27].	Clinical examination: FAST criteria. Imaging: CT/MRI to differentiate stroke types. Blood tests: Identify risk factors. ECG/Echocardiogram: Heart‐related causes [28].
Epilepsy	Seizures: Generalized tonic‐clonic (convulsions) or focal seizures [29]. Aura: Sensory changes or unusual emotions before a seizure [31].	Seizure frequency and severity can increase without treatment. The impact on quality of life varies [30].	Clinical history: Seizure type, frequency, triggers. EEG: Detects abnormal brain activity. Neuroimaging: MRI/CT for structural abnormalities [32].
Parkinson's disease	Motor symptoms: Tremor, bradykinesia, rigidity, postural instability. Non‐motor symptoms: Cognitive decline, mood disorders, sleep disturbances, autonomic dysfunction [33].	Progressive worsening of symptoms, leading to significant disability over time.	Clinical examination: Based on motor symptoms and response to dopaminergic therapy. Imaging: DaTscan (if needed) to confirm dopamine deficiency [34].
Meningitis	Bacterial meningitis: Sudden fever, headache, neck stiffness, photophobia, nausea, vomiting. Viral meningitis: Similar but milder symptoms, often with flu‐like onset [35].	Bacterial meningitis can be rapidly fatal or cause severe complications. Viral meningitis has a better prognosis but can cause prolonged recovery [36].	Lumbar puncture: CSF analysis. Blood cultures: Identify causative organisms (bacterial meningitis). Imaging: CT/MRI if complications are suspected [35].

## 6 Treatment and Management

Systemic problems in Bangladeshi healthcare, such as restricted access to specialist care, diagnostic equipment, and pharmaceuticals, pose serious hurdles to the treatment and management of neurological illnesses. Poor surgical facilities and rehabilitation services jeopardize the care of hemorrhagic stroke patients, whereas delayed hospital admission and outdated imaging technologies impede the treatment of ischemic stroke patients in particular [37].

Antiepileptic pharmaceutical therapy of epilepsy is successful, although it is hampered by high costs, social stigma, and recurrent supply shortages [32]. Treatment for Parkinson's disease is hampered by long‐term side effects and the lack of cutting‐edge treatments such as deep brain stimulation, which is mostly achieved with levodopa [38]. Timely delivery of antibiotics is crucial for the successful treatment of meningitis; however, results might be affected by delayed diagnosis and uneven vaccination coverage, particularly in rural areas [39].

Sociocultural hurdles, financial limitations (such as high treatment costs and scant insurance coverage), and differences in healthcare access between urban and rural areas all compound these issues. To guarantee more fair and efficient care for neurological patients in Bangladesh, addressing these concerns calls for advancements in the country's healthcare system, pharmaceutical accessibility, and public awareness campaigns.

## 7 Public Health Implications

**Figure 3:**
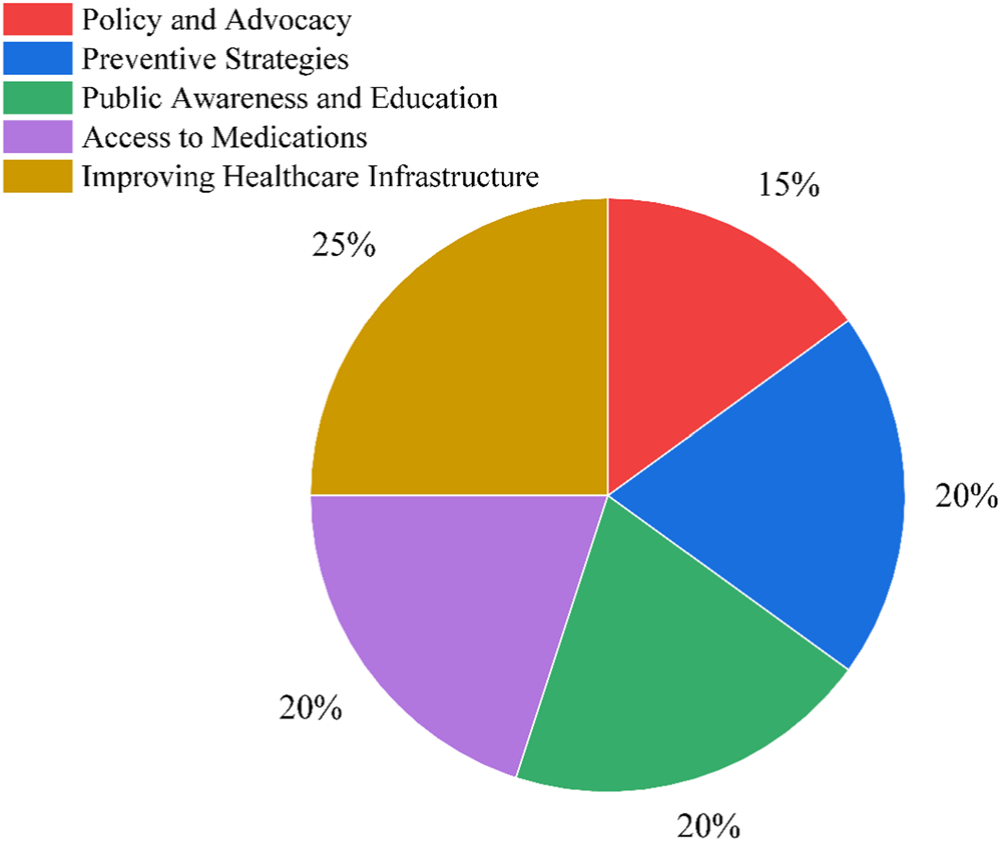
Strategies for prevention and control of neurological diseases in Bangladesh.

Bangladesh has a high prevalence of neurological illnesses, with serious public health consequences associated with disorders including meningitis, stroke, epilepsy, and Parkinson's disease. Particularly in groups who are already at risk, these illnesses greatly increase disability, death, and medical expenses [16]. The rising incidence of these illnesses, which are made worse by poor infrastructure, a lack of specialist treatment, and restricted access to essential drugs, is placing a tremendous burden on the healthcare system. Due to delays in diagnosis and treatment, many individuals experience extended impairment and avoidable consequences, which has a devastating impact on the population [17]. Figure 3 shows the significance of using a multimodal strategy for treating neurological illnesses in Bangladesh, including public awareness campaigns, pharmaceutical availability, infrastructure improvements in the healthcare system, preventative measures, and policy lobbying.

The largest part, which accounts for 25% of the total, emphasizes the necessity of improving healthcare infrastructure, emphasizing the significance of constructing better facilities, outfitting hospitals, and guaranteeing access to expert neurological treatment. To lower the incidence of disease, 20% of the focus is on preventive measures, which emphasize the significance of reducing modifiable risk factors including smoking, diabetes, high blood pressure, and environmental pollution [42, 49]. An additional 20% goes toward education and public awareness, which emphasizes the need to educate people about neurological illnesses' early warning signs and lowering the stigma attached to diseases like epilepsy and Parkinson's disease. The availability of pharmaceuticals is also emphasized as a crucial element, contributing 20% of the endeavors to guarantee patients in both urban and rural regions have access to reasonably priced and efficient treatment alternatives. The lowest portion, 15%, is devoted to lobbying and policy, which aims to integrate neurological health into larger national health plans, increase financing, and create supportive healthcare regulations [50, 51]. With an emphasis on prevention, infrastructure upgrades, education, medicine accessibility, and policy developments, the Bangladeshi government is tackling the rising prevalence of neurological disorders in a balanced manner. Research highlights the importance of awareness campaigns, prevention, and infrastructure limitations in the treatment of neurological illnesses. A diversified strategy is needed to address this load. Providing prompt, specialized care requires improved healthcare infrastructure, particularly in rural locations. It is also crucial to provide regular access to necessary pharmaceuticals and lower their cost. Campaigns for early intervention and public awareness can help lessen stigma, especially for illnesses like epilepsy [40]. The management of these conditions depends on preventive measures, such as meningitis immunization campaigns and lifestyle modifications to lower the risk of stroke. The fight to lessen the prevalence of neurological illnesses in Bangladesh might be aided by promoting stricter laws and more international cooperation.

## 8 Conclusion

Given the high rates of disease burden, disability, and mortality in Bangladesh, neurological disorders represent a serious public health concern. Important illnesses like meningitis, stroke, epilepsy, and Parkinson's disease are made worse by a lack of neurologists, socioeconomic inequalities, and inadequate healthcare resources. Addressing these challenges requires improving healthcare infrastructure, especially in rural areas, ensuring access to affordable medications, and launching public education campaigns to reduce stigma and promote early intervention. Stronger policies, preventive measures, and international collaboration are essential to improving neurological health and overall well‐being in the country.

## Author Contributions

**Mst. Mohona Khatun:** investigation, software, data curation, writing – review and editing, writing – original draft, validation, visualization. **Mohammad Shahangir Biswas:** conceptualization, methodology, investigation, software, data curation, supervision, project administration, writing – review and editing, writing – original draft, validation, visualization. **Suronjit Kumar Roy:** writing – review and editing. **Md Foyzur Rahman:** writing – review and editing, resources. **Rubait Hasan:** writing – review and editing. **Syed Masudur Rahman Dewan:** writing – review and editing. **Munna Kumar Podder:** writing – review and editing. All authors have read and approved the final version of the manuscript.

## Ethics Statement

The authors have nothing to report.

## Conflicts of Interest

The authors declare no conflicts of interest.

## Transparency Statement

The lead author, Mohammad Shahangir Biswas, affirms that this manuscript is an honest, accurate, and transparent account of the study being reported, that no important aspects of the study have been omitted and that any discrepancies from the study as planned (and, if relevant, registered) have been explained.

## Data Availability

The data that support the findings of this study are available on request from the corresponding author. The data are not publicly available due to privacy or ethical restrictions. [Corresponding author or Manuscript guarantor] had full access to all of the data in this study and takes complete responsibility for the integrity of the data and the accuracy of the data analysis.
